# Multiplex Photoluminescent Silicon Nanoprobe for Diagnostic Bioimaging and Intracellular Analysis

**DOI:** 10.1002/advs.201700548

**Published:** 2017-12-31

**Authors:** Meysam Keshavarz, Bo Tan, Krishnan Venkatakrishnan

**Affiliations:** ^1^ Nanocharacterization Laboratory Department of Aerospace Engineering Ryerson University 350 Victoria Street Toronto ON M5B 2K3 Canada; ^2^ Institute for Biomedical Engineering Science and Technology (iBEST) Partnership between Ryerson University and St. Michael's Hospital Toronto ON M5B 1W8 Canada; ^3^ Ultrashort Laser Nanomanufacturing Research Facility Department of Mechanical and Industrial Engineering Ryerson University 350 Victoria Street Toronto ON M5B 2K3 Canada; ^4^ NanoBioInterface Facility Department of Mechanical and Industrial Engineering Ryerson University 350 Victoria Street Toronto ON M5B 2K3 Canada; ^5^ Keenan Research Centre for Biomedical Science St. Michael's Hospital Toronto ON M5B 1W8 Canada

**Keywords:** bioimaging, diagnosis, fibroblasts, HeLa, multiplex photoluminescence

## Abstract

Herein, a label‐free multiplex photoluminescent silicon nanoprobe (PLSN‐probe) is introduced as a potential substitute for quantum dots (QDs) in bioimaging. An inherently non‐photoluminescent silicon substrate is altered to create the PLSN‐probe, to overcome the major drawbacks of presently available QDs. Additionally, crystallinity alterations of the multiplane crystalline PLSN‐probes lead to broad absorption and multiplex fluorescence emissions, which are attributed to the simultaneous existence of multiple crystal planes. The PLSN‐probe not only demonstrates unique optical properties that can be exploited for bioimaging but also exhibits cell‐selective uptake that allows the differentiation and diagnosis of HeLa and fibroblast cells. Moreover, multiplex emissions of the PLSN‐probe illuminate different organelles such as the nucleus, nucleolemma, and cytoskeleton, depending on size‐based preferential uptake by the cell organs. This in vitro study reveals that cancerous HeLa cells have a higher propensity for taking up the PLSN‐probe compared to fibroblast cells, allowing the diagnosis of cancerous HeLa cells. Additionally, the fluorescence intensity per unit area of the cell is found to be a reliable means for distinguishing between dead and healthy cells. It is anticipated that the multifunctionality of the PLSN‐probes will lead to better insight into the use of such probes for bioimaging and diagnosis applications.

## 1 Introduction

Fluorescence (FL) microscopy is extensively used as a crucial tool for biological applications such as biomedical imaging and clinical diagnostics.^1^ However, conventional cell imaging techniques that utilize organic fluorophores have some major problems due to their nonspecific accumulation within cells and photobleaching of the dyes.^2^, ^3^ Commercially available organic dyes have been routinely used in life sciences for FL imaging; however, these dyes have some inadequacies that hinder them in the rapidly growing field of bioimaging. The main drawbacks include a limited lifetime (a few nanoseconds), progressive reduction of FL (photobleaching), low contrast in some applications, emission spectra with a red tail, and low emission times. Hence, an imaging method that is not reliant on fluorophore dyes is needed.^4^ Therefore, extensive efforts have been made to produce an alternative to organic fluorescent molecules to alleviate these problems.^5^

In particular, recent research has highlighted the feasibility of using quantum dots (QDs) as optically efficient alternatives to fluorescent dyes for bioimaging applications. However, the applicability of QDs for live cell imaging has been severely restricted because of cytotoxicity problems, blinking, particle size dependency, and reliance on bioconjugation for delivery. Hence, methods for labeling as well as biocompatible coatings are essential for QDs.^3^, ^6^, ^7^

A wide variety of quantum‐scale materials have been studied for diverse bioimaging usage; among them, semiconductor quantum dots (silicon‐based QDs), noble nanoparticles (gold), super‐paramagnetic oxides (iron oxide), and carbon materials Single‐walled carbon nanotubes (SWCNT) have been extensively exploited for optical bioimaging purposes.^8^, ^9^ As strong contrast agents, these nanoscale materials possess a range of advantages over conventional organic‐based fluorescent dyes, including a higher stability in terms of photostability and broader excitation and narrower emittance wavelengths. For instance, cadmium selenide (CdSe) QDs have been widely used due to their broad range of emitted colors, which are tunable based on their size.^10^ However, the cytotoxicity of QDs doped with heavy metallic constituents such as cadmium remains a serious concern.^11^ To address this issue, a surface postmodification process is required to obtain biocompatible doped QDs. Despite surface modifications of the QDs, the inherent cytotoxicity of doped QDs has hampered their clinical application.^12^ Although numerous studies have been performed to coat the toxic cores of QDs containing II–VI metallic groups, safety concerns have not yet been completely overcome, and the inherent cytotoxicity of noble QDs is one of the major obstacles for their further clinical application.^13^

The surface chemistry of Si‐based QDs (Si‐QDs) is of great interest because of their considerable difference from heavy metal QDs. In addition, the utility of Si‐QDs in bioimaging lies in their optical properties, which are size‐ and crystallinity‐dependent. Si‐QDs have shown to be a substantial breakthrough as a bioimaging agent due to their nontoxicity and biodegradability; one byproduct of degradable Si‐QDs, silicic acid, can even be readily excreted via the urine.^14^ Although Si‐QDs have drawn attention due to their superior biocompatibility, key challenges of high quantum yield and long‐term stability in water and biological media must be overcome.^15^ Additionally, the delivery of Si‐QDs is dependent on bioconjugation.^16^ Numerous studies have been reported on the targeted usage of bioimaging agents. For instance, Tilley et al. demonstrated nonspecific uptake of allylamine‐terminated blue‐emitting Si‐QDs in HeLa cells.^17^ Montalti et al. have reviewed the recent progress on employment of Si‐QDs as an ultrastable and biocompatible probe for luminescent bioimaging, and concluded the superior stability of the Si‐QDs to in physiological environments such as in vitro and in vivo with no photobleaching deterioration and unique biocompatibility.^18^ Reipa and co‐workers' laboratory conjugated red‐emitting silicon nanoparticles (Si‐NPs) to streptavidin and demonstrated specific binding of the nanoparticles to biotinylated polystyrene beads.^19^ Despite progress in bioconjugation chemistry, the size‐tunable luminescence characteristics, as well as the minimal autofluorescence of the Si‐QDs present challenges.^20^ One of the customary coatings of QDs, mercaptoacetic acid, has been found to be cytotoxic.^21^ The exposed metallic core of coated QDs due to dissolution of the coating can also be toxic.^22^ Cadmium and selenium ions, which are used in the core of QDs, are known to be cytotoxic.^23^ In addition, erosion of the shell may cause undesirable reactions in vivo.^24^ QDs may be toxic based on their composition, as reported in in vitro studies.^25^ CdSe QDs coated with mercaptoacetic acid were found to be toxic to rat pheochromocytoma cells.^26^ The mechanism of cell death is unknown, but it is believed to be caused by the free Cd released by core degradation.^27^

Moreover, QDs are highly sensitive to surface defects, which can reduce the quantum yield of QDs by affecting the recombination of electrons and holes, resulting in blinking of the QDs.^28^ This flaw, however, can be diminished by coating a shell around the QD.^29^ QDs have shown a strong tendency to aggregate when placed in live cells, which interferes with cell function.^17^ A difficulty also exists in delivering QDs inside cells without killing and/or damaging the cells in the process.^19^ Hence, conjugation of the QDs with different biomolecules or biolabels is required.^30^ Although QDs are in the nanometer size range, bioconjugation increases their size, rendering their delivery into cells more difficult.^31^

Here, we report the first development of multiplex emissions from photoluminescent silicon nanoprobes (PLSN‐probes). The transition from a single (polyhedron) to multiplanar (polyhedral) silicon crystal structure yields a higher absorption and hence a superior FL throughput. Unlike QDs, the PLSN‐probes not only exhibit superior optical properties including higher brightness and narrower emission and broader absorption spectra, but also cell‐selective uptake, which eliminates the need for coating and bioconjugation. This unique nanoprobe emits a higher FL intensity when there is a greater number of crystal planes (polyhedral PLSN‐probes), and it is hypothesized that the multicrystal orientation is the cause of the multiplex emission of the PLSN‐probes, which has never been observed with QDs.

The use of ultrashort pulsed laser (USPL)‐assisted ionization to synthesize PLSN‐probes under inert atmospheric conditions resulted in impurity‐free and well‐characterized 3D structures. The PLSN‐probes were created based on kinetic self‐assembly activated by multiphoton ionization. By using USPL, we were able to systematically transform a nonluminescent bulk single‐crystal silicon wafer into multiplex photoluminescent nanoprobes with tunability via synthesized crystal orientations.^32^ The higher number of concurrent crystal planes increases the absorption and subsequently the FL emission of the PLSN‐probes. The wide‐range absorption and multiplex narrow emittance of the PLSN‐probes, wavelength‐dependent phenomena, were exploited to illuminate different organs of the cells. Internalization of the PLSN‐probes into cells allowed intercellular observation at the distinct excitation wavelength, demonstrating that the functions of multiple dyes can be simultaneously mimicked by PLSN‐probes. The size distribution of the synthesized PLSN‐probes was regulated to dimensions in which the quantum confinement effect is preeminent by precisely tuning the laser fluence. The smaller‐sized Si‐NPs, ranging between 2 and 2.6 nm, show an affinity for internalization, allowing the cell nucleus and nucleolemma to be discerned. PLSN‐probes measuring 3.5 nm were also permeable through the cell membrane and were able to indicate details of external organs such as the cytoskeleton, microtubules, and lamellipodium. Additionally, this versatile method of bioimaging has shown diagnostic competency through which HeLa cancer cells can be differentiated from mammalian fibroblast cells. Moreover, dead and viable cells can be screened by a comparison of emitted intensities. The PLSN‐probes showed excellent biocompatibility and high photostability within live cells for longer periods compared to fluorescent dye alone. The biocompatibility and photostability of the PLSN‐probes were examined after 24 and 48 h of incubation on two HeLa and fibroblast cell lines. Notably, this is the first report on the use of PLSN‐probes as a cell‐selective uptake bioimaging agent with the direct possibility of diagnosing HeLa cells as well as a general efficiency for bioimaging.

## 2 Results and Discussion

### 2.1 Fabrication and Structural Characterization

The PLSN‐probes were synthesized via USPL‐assisted ionization. The absorption of one or more photons by electrons in atoms of the substrate (a single‐crystal silicon wafer) causes spatial electron separation of the excited atom or ion species. To prevent any compositional changes, this process was carried out in the presence of an inert gas. The USPL has shown a capability of overcoming the ionization threshold of the silicon substrate by inducing sufficient photon energy. The accumulated energy of multiphoton absorption exceeds the ionization potential of the silicon and hence leads to atomic ionization. Therefore, the kinetics of the self‐assembly process were initiated by multi‐photoionization. The experiment showed that multi‐photoionization can be governed by precisely tuning the laser fluence. When a sufficiently higher laser fluence interacts with the substrate, it results in the formation of a 3D self‐assembled structure consisting of multiplanar crystal Si (polyhedral). Likewise, a lower laser fluence resulted in fewer crystal planes (polyhedron). The synthesis of the PLSN‐probes can be precisely tuned by regulating the laser parameters. While the laser pulse duration and laser power were kept constant at 214 fs and 16 W, respectively, high, intermediate, and low laser fluences were applied at a 4, 12, and 26 MHz repetition rate to synthesize polyhedron, intermediate, and polyhedral PLSN‐probes. Based on the polycrystallinity of the synthesized probe, they categorized in polyhedron, intermediate, and polyhedral PLSN‐probes.

**Figure 1:**
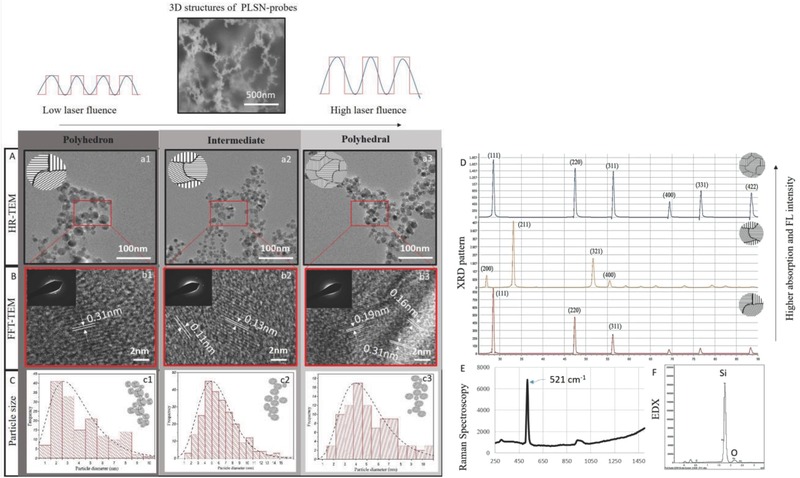
Characterization of the PLSN‐probes. A) HR‐TEM images illustrating (a1) polyhedron, (a2) intermediate, and (a3) polyhedral PLSN‐probes. B) Crystal *d*‐spacing and diffraction are demonstrated in images (b1–b3). C) Size distribution of the fused PLSN‐probes plotted in histograms (statistical analysis performed over 1000 random particles with an standard deviation (SD) of 0.7). D) XRD patterns show the dominance of multiple crystal planes upon increased laser fluence. E) Raman spectroscopy demonstrates the formation of crystalline PLSN‐probes. F) EDX results acquired from the synthesized PLSN‐probes do not indicate any compositional changes.

Transmission electron microscopy (TEM) analysis (**Figure** **1**A) was carried out to study the crystallinity and size distribution of polyhedron (Figure 1(a1)), intermediate (Figure 1(a2)), and polyhedral PLSN‐probes (Figure 1(a3)). High‐resolution transmission electron microscope (HR‐TEM) images (Figure 1A) show fused sub‐nanospherical silicon particles with a narrow size distribution in the range of 2–10 ± 0.7 nm (Figure 1C). The presence of multiple crystal orientations in the polyhedron, intermediate, and polyhedral PLSN‐probes was also observed on fast Fourier transform (FFT)–HR‐TEM images (Figure 1B). The alteration of the crystal planes, along with the maintained size distribution range, rules out the influence of size range on the further optical characteristics of the as‐synthesized PLSN‐probes.

Additionally, X‐ray diffraction (XRD) analysis (Figure 1D) further confirms the dominance of 3, 4, and 6 multiple crystal planes upon increased laser fluence. Chemical and elemental analyses of the PLSN‐probes were carried out by energy‐dispersive X‐ray spectroscopy (EDX) (Figure 1E), where no trace of chemical changes was detected. EDX confirmed the presence of pure silicon. Furthermore, as depicted in Figure 1F, the Raman peak at 521 cm^−1^, which is the characteristic of crystalline silicon, has been identified in all the synthesized PLSN‐probes. All of the evidences demonstrate the successful formation of pure 3D structured PLSN‐probes with varied crystal planes at the same size distribution range.

#### 2.1.1 Optical Characterization

**Figure 2:**
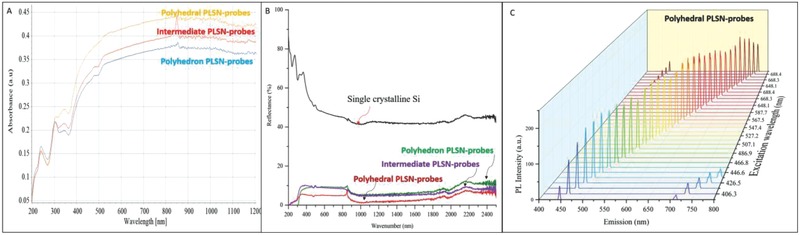
Optical properties of the PLSN‐probes with respect to crystal plane variation. A) UV–vis–NIR spectra of the PLSN‐probes exhibit an increase in photon absorption with polyhedral PLSN‐probes in the vis and NIR regions. As the number of crystal planes increases, the absorption increases. B) Reflectance spectra of the PLSN‐probes. The black color spectrum corresponds to the silicon substrate (reference). The PLSN‐probes at different ranges of particle size show relatively the same reflection/absorption. The reflectance over the entire UV and NIR band is lowered. PLSN‐probes show significantly higher optical absorption, especially in the UV and NIR regions. C) Room‐temperature photoluminescence (PL) spectra of the polyhedral PLSN‐probes.

**Figure 3:**
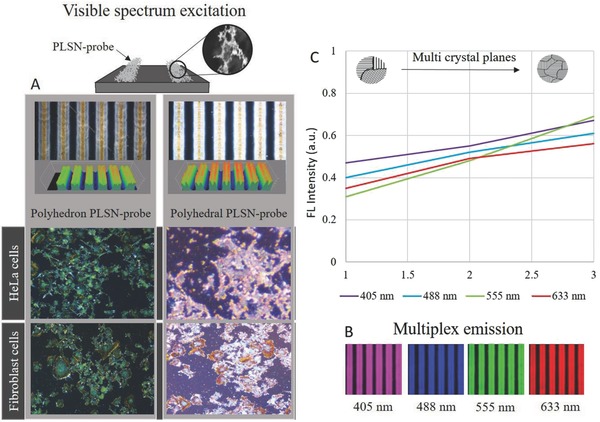
Influence of the multicrystal planes on PLSN‐probes. A) Irradiation at the visible spectrum shows a stronger excitation of the polyhedral PLSN‐probes, also brightfield microscopy of HeLa and fibroblast exhibit higher intensity of polyhedral PLSN‐probes. B) Emission of the polyhedral PLSN‐probes at excitation wavelengths of 405, 488, 555, and 633 nm exhibits the multiplex characteristics of the PLSN‐probes. C) Changes in normalized FL intensity due to crystallinity alteration of the PLSN‐probes are shown. A comparison of florescence intensity at excitation wavelengths of 405, 488, 555, and 633 nm shows the higher emission intensity of the polyhedral PLSN‐probes.

The PLSN‐probes were first investigated in terms of ultraviolet–visible–near‐infrared (UV–vis–NIR) reflection and absorption at room temperature, as shown in **Figure** **2**A,B. Compared to the bulk silicon, irradiation of the PLSN‐probes ranging from 200 to 1200 nm exhibited a significantly lower reflection, which can be translated to high photon absorption, as shown in Figure 2B. However, higher absorption was observed for the polyhedral PLSN‐probes. The transformation of single‐crystal silicon (substrate) to polycrystalline PLSN‐probes at different ionization levels resulted in an alteration of the dominant crystal planes and, ultimately, an enhancement in absorption properties (Figure 2A). The UV–vis–NIR spectroscopy revealed that strong absorption occurs in the polyhedral PLSN‐probes. This higher absorption is attributed to the distinct photon absorption of each available crystal plane. In particular, lower absorption was observed at a lower wavelength of the UV region, whereas upon irradiation of vis light (≈400–700 nm), the synthesized PLSN‐probes showed higher absorption and stunted reflection. However, all the three types of synthesized probes exhibited multiplex emission due to their polycrystallinity. The polyhedral probes were chosen due to the higher absorption and FL intensity compared to intermediate and polyhedron probes (less polycrystallinity). Figure 2C presents the photoluminescence (PL) spectra of the polyhedral PLSN‐probes at room temperature. All of the samples showed strong PL with respect to the excitation wavelength. The emission at a longer wavelength and hence lower intensity demonstrate the FL excitation of the PLSN‐probes; notably, broad absorption and narrow emission. As the excitation wavelength increased, the emission shifted toward a longer wavelength. However, the FL intensity decreased at wavelengths longer than 700 nm. The overall narrow full width at half maximum and long Stokes shifts are the two major PL characteristics that are significant for successful application of the PLSN‐probes for bioimaging.^33^ Since the PLSN‐probe consists of fused particles ranging from 2 to 10 nm, broader absorption and a subsequent wide range of emission were achieved. The FL intensity showed an 80% increase as the excitation wavelength shifted toward 600 nm, after which the FL intensity decreased and the emission color was gradually redshifted. Moreover, irradiation of the PLSN‐probes in the visible range, shown in **Figure** **3**A, demonstrated higher absorption of the polyhedral PLSN‐probes compared to the intermediate and polyhedron PLSN‐probes which was also observed on brightfield microscopy of both HeLa and fibroblast cells. However, all the probe types exhibited multiplex emissions at excitation wavelengths ranging from 405 to 633 nm, as shown in Figure 3B. A plot of the normalized FL intensity (Figure 3C) reveals significantly higher emission intensity of the polyhedral PLSN‐probes. Hence, the polyhedral PLSN‐probes were chosen for further bioimaging studies. Strong changes in FL emission are not caused by conjugated lengths, making the PLSN‐probes suitable for bioapplications in which the toxicity of fluorescent agents and their lengths are primary concerns.

The results imply that by varying the excitation wavelength, different FL emissions ranging from 500 to 800 nm can be achieved. Furthermore, for excitation at a longer wavelength, a redshift emission at 650 nm can be interpreted from the excitation and emission profile. Since the FL domain of the PLSN‐probes is wavelength‐dependent, the presence of a diverse size distribution was neutralized by narrowing the excitation wavelength. This phenomenon also revealed that size variations in the PLSN‐probes may allow one to precisely target different organs of the cell, which can then be distinguished at distinct excitation wavelengths. Figure 3C depicts a higher FL peak intensity observed with polyhedral PLSN‐probes at four excitation wavelengths (405, 488, 555, and 633 nm) commonly used in bioimaging. Moreover, fluorescence quantum yields exceeding 74% have been achieved at grater polycrystallinity (polyhedral PLSN‐probes). The spectral features of the polyhedral PLSN‐probes tabulated in **Table** **1** elucidate the emissions acquired for bioimaging of HeLa and fibroblast at corresponding excitation wavelengths.

**Table 1:** The spectral features of the polyhedral PLSN‐probes. The emission acquired for bioimaging at the excitation wavelength of 405, 488, 555, and 633 nm, respectively, are 447, 525, 617, and 635 nm (longpass)

Excitation wavelengths [nm]				Included emission filters	
UV	Blue	Green	Red	Emission filters (center wavelength/bandwidth)	Dichroic wavelength [nm]
405	488	555	633	447 nm/60 nm, 525 nm/45 nm, 617 nm/36 nm, and 635 nm/longpass	495, 562, and 469

The acquired result from excitation and emission spectra fits well with observations from confocal FL microscopy. The pronounced variational excitation features evince that the PLSN‐probes are indeed multiplex emissive and wavelength‐dependent. These attributes may be useful in exploring the implementation of polyhedral PLSN‐probes for bioimaging.

**Figure 4:**
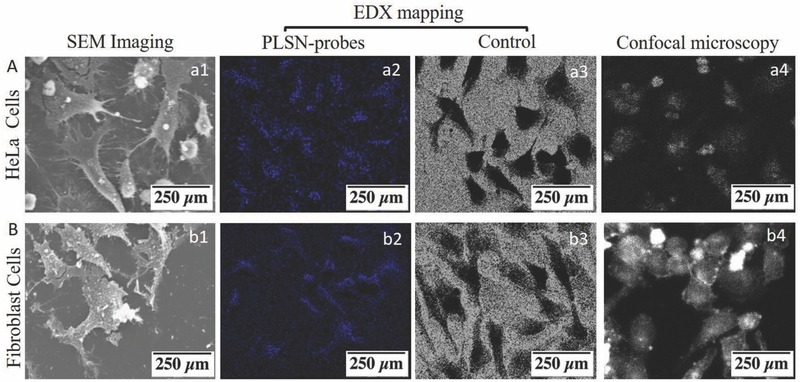
PLSN‐probe Internalization into cytoplasm of A) HeLa and B) fibroblast. SEM images of HeLa (a1) and fibroblast (b1) in presence of the PLSN‐probes. EDX elemental mapping of silicon (blue color) shows the distribution of the PLSN‐probes within the HeLa (a2) and fibroblast (b2). Control EDX images in absence of the PLSN‐probes for HeLa (a3) and fibroblast (b3). Confocal imaging shows the illumination of uptake probes in bright spots of fibroblast and HeLa (a4, b4), respectively.

Elemental mapping of HeLa and fibroblast in presence of polyhedral PLSN‐probes, as shown in **Figure** **4**A,B, respectively, elucidates the internalization of the Si‐based probes within the HeLa and fibroblast (a2 and b2) compared to the control (a3 and b3), where no trace of silicon was found due to the absence of the PLSN‐probes. Additionally, confocal microscopy revealed bright spots representing the illumination of the PLSN‐probes as depicted in Figure 4(a4, b4). However, due to the permeability differences in membrane of fibroblast (Figure 4(b4)) and HeLa (Figure 4(a4)), the PLSN‐probes illuminate the cytoskeleton of fibroblast whereas they are well‐spread within the HeLa cells. Taken together, the EDX and confocal microscopy results from HeLa and fibroblasts led to the conclusion that the PLSN‐probes infiltrate through the cell membranes.

### 2.2 Bioimaging and Intercellular Analysis

**Figure 5:**
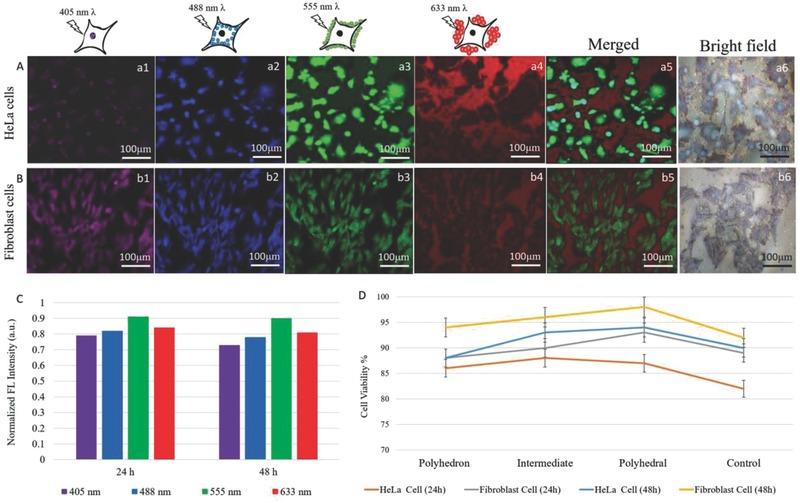
Cell‐selective uptake of the PLSN‐probes. Confocal microscopy images of A) HeLa and B) fibroblast cell lines without any fluorophores. Image of the cells after incubation with PLSN‐probes at 405, 488, 555, and 633 nm revealed cell‐selective uptake. HeLa cells exhibited sequential uptake (a1–a4) through which different layers of the cell, including the cytoskeleton, nucleolemma, and nucleus as well as the cell periphery, were screened. Images of fibroblast cells (b1–b4) imply an aversion toward cellular uptake as only the cytoskeleton of the cells could be discerned. Merged images taken at different excitation wavelengths display intercellular details of HeLa cells (a5) but only the cell periphery and cytoskeleton of the fibroblast cells (b5). Brightfield images of HeLa (a6) and fibroblast (b6). C) The FL intensity of the multiplex PLSN‐probes after 24 and 48 h of incubation revealed negligible intensity decay. D) A cell viability assay demonstrated superior biocompatibility of the PLSN‐probes after 48 h on both HeLa and fibroblast cells.

We investigated the possible use of PLSN‐probes for optical imaging of cancerous HeLa and mammalian fibroblast cells. Confocal FL microscopy of the HeLa and fibroblast cells at four excitation wavelengths, 405, 488, 555, and 633 nm (**Figure** **5**A,B), revealed the multiplex attribution of the PLSN‐probes, by which the probes can be excited at different wavelengths. This characteristic was exploited to illuminate different parts of the cell, each at a distinct wavelength.

The self‐internalization of the PLSN‐probes is impelled by adsorption of proteins upon dispersion in the cell culture medium.^34^ The quantum‐scale of the PLSN‐probes not only provide extended surface area, which is ideal to adsorb the additive proteins in the serum, but also drives the infiltration through the cell membrane.^9^, ^35^, ^36^ The in vitro uptake of the PLSN‐probes by living cells was studied to identify the location of the internalized PLSN‐probes within the cells. Strikingly, HeLa cells exhibited selective uptake, as shown in Figure 5(a1–a4). As the PLSN‐probe internalizes through the cell membrane, it breaks apart within the cell body and further penetrates into the different layers of the cell. However, infiltration of the PLSN‐probes is dependent on permeability differences of the plasma membrane and nucleolemma of a cell.^9^, ^36^, ^37^

The purple emission at the 405 nm excitation wavelength of the PLSN‐probes was primarily observed from the nuclear region, while the blue and green emissions, excited at 488 and 555 nm, correspond to the nucleolemma and cytoskeleton of the cell. The red emission displayed PLSN‐probes that had not been taken up and, therefore, showed only the periphery of cells. In this case, however, this phenomenon was found to be restricted to the HeLa cells. Figure 5A depicts the sequential uptake penetration of the PLSN‐probes. Because the emission of shorter wavelengths correlated to the size of the probes, the ability to visualize the nuclei at 405 nm implies that smaller PLSN‐probes had penetrated the cell nuclei. Likewise, the nucleolemma and cytoskeleton were similarly distinguished. Our results reveal that the PLSN‐probes of ≈2 nm tended to infiltrate the nuclei, whereas the slightly larger PLSN‐probes of ≈2.6 nm surrounded the nucleolemma. Similarly, the 3.5 nm PLSN‐probes were shown to be attracted to the cytoskeleton–membrane. The 6.2 nm PLSN‐probes could not permeate the cell membrane, and hence, these PLSN‐probes, excited at 633 nm, displayed the periphery of the cells. However, as depicted in Figure 5(b1–b4), the uptake differed in the fibroblast cells for this imaging agent, and only the cytoskeleton could be visualized at all four tested wavelengths. Hence, the image (Figure 5(b5)) depicted overlay of red and green which are the higher intensity as plotted in Figure 5C. Additionally, the brightfield images of HeLa and fibroblast in presence of the PLSN‐probes demonstrated in Figure 5(a6, b6), respectively. The cell‐selective uptake is hypothesized to be due to the difference in permeability of these two cell lines, leading to the potential use of the PLSN‐probes for diagnosis of HeLa cells. The limited uptake of the PLSN‐probes by the fibroblast cells can be exploited to distinguish between HeLa and fibroblast cells. In addition, the biocompatibility of the PLSN‐probes was assessed after 24 and 48 h of incubation. A cell viability assay (Figure 5D) showed the superior biocompatibility of the PLSN‐probes. The increase on the cell viability exhibited after 48 h for both HeLa and fibroblast is because of proliferation factor that increases over the course of cell culture after which HeLa cells demonstrated clustering and fibroblasts tend to form tissue like structures. The FL intensity of the PLSN‐probes was also examined over the course of 24 and 48 h to rule out the influence of gradual disintegration; as shown in Figure 5C, negligible differences and exceptionally strong FL intensities were observed during the 48 h of incubation.

**Figure 6:**
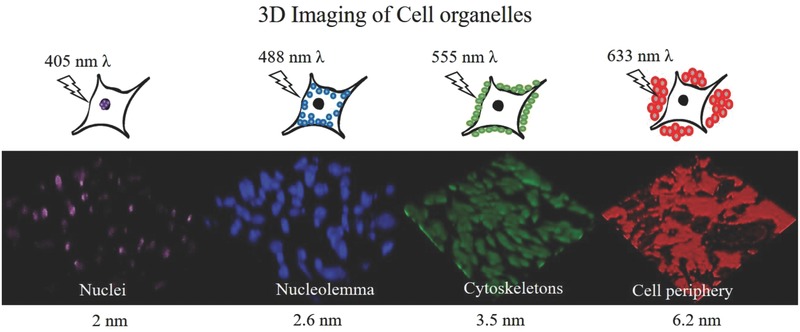
Intracellular analysis of the HeLa cells. Selective uptake of the PLSN‐probes made it possible to visualize intercellular organs of individual cells. From left to right: nuclei, nucleolemma, cytoskeleton, and the cell periphery were depicted by internalization of the PLSN‐probes.

**Figure** **6** shows the 3D visualization of intracellular organs of HeLa cells by confocal FL microscopy. These images provide details regarding the uptake and aggregation of the PLSN‐probes within a cell. To the best of our knowledge, this is the first report on the use of PLSN‐probes that are cell selective and possess a multiplex characteristic for 3D imaging of live cancer cells. The EDX elemental mapping (Figure 4) is in accordance with the confocal microscopy images demonstrated in Figure 5A,B, where these PLSN‐probes did not have a tendency to aggregate within the cytoplasm, which would cause cellular damage as well as lessen visibility. The agglomeration of nanoscale particles and nonspecific accumulation of organic dyes are common drawbacks of these imaging agents but has not been observed with the PLSN‐probes.^38^

**Figure 7:**
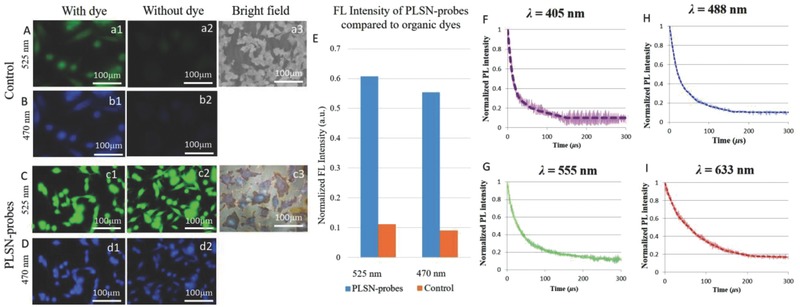
PLSN‐probes for FL bioimaging. A–D) FL imaging of cells on the control sample and in the presence of PLSN‐probes, respectively. (a3 and c3) demonstrate the brightfield images corresponding to the (A, B) and (C, D). E) The intensity of emitted spectra plotted illustrates the remarkable FL intensity of the PLSN‐probes compared to the control without dyes. F–I) In vitro normalized photoluminescence lifetime curve of simultaneously recorded microsecond‐decaying of PLSN‐probes and the corresponding fits (dashed line) at 405, 488, 555, and 633 nm excitation wavelengths are demonstrated.

To determine the application prospects of these novel PLSN‐probes as efficient bioimaging tools, they were compared to two commonly used dyes, 4′,6‐diamidino‐2‐phenylindole (DAPI) and fluorescein isothiocyanate (FITC) (control), in the absence of the PLSN‐probes, as shown in **Figure** **7**A,B. The cells were prepared for FL imaging by adding a small aliquot of the PLSN‐probes (without the addition of any dyes) to aqueous cell culture medium, followed by fixation. The results are demonstrated in Figure 7(c2, d2). Cells stained with DAPI and FITC in the presence of PLSN‐probes displayed brighter specification (Figure 7(c1, d1)) relative to the control (Figure 7(a1, b1)). Brightfield images of control and in presence of the PLSN‐probes are depicted in Figure 7(a3, c3), respectively. The higher FL intensity of the PLSN‐probes compared to DAPI and FITC was measured, as shown in Figure 7E. Comparison of the FL microscopy of samples stained with dyes and those supplemented with PLSN‐probes shows a significant difference in which control cells are not distinguishable (Figure 7(a2, b2)). In contrast, the PLSN‐probes strongly enhanced the FL imaging and emitted in the visible region when excited at both 470 and 525 nm, as shown in Figure 7(c2, d2). Therefore, as is evidenced in Figure 6, the PLSN‐probes dispersed in cell culture medium can be substituted for organic dyes. Additionally, quantitative assessment of their FL intensities (Figure 7E) implies a higher emission intensity of FITC and DAPI dyes when assisted with the PLSN‐probes as compared to samples stained with only FITC and DAPI dyes. The obtained in vitro PL decay lifetime of polyhedral PLSN‐probes is exhibited in Figure 7F–I, respectively, at 405, 488, 555, and 633 nm wavelengths. The PL decay lifetime of the synthesized probes was found to be independent on the cell type and yet wavelength related, where broadening the wavelength from 405 to 633 resulted in extended lifetime of the probes. However, as depicted in Figure 5C, the 555 nm wavelength (green spectrum) has higher FL intensity.

**Figure 8:**
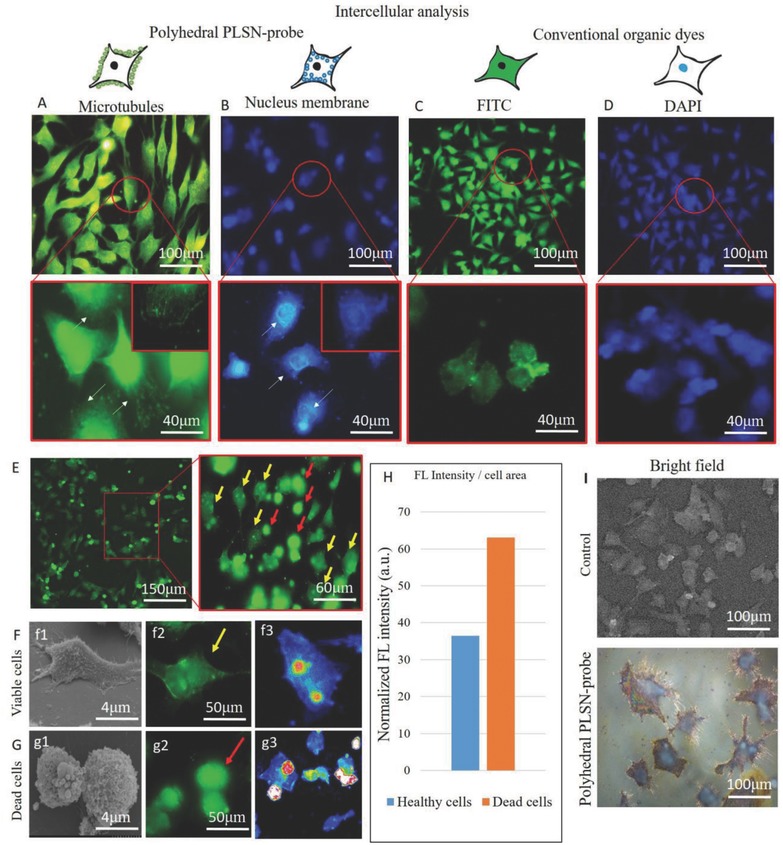
A) Intercellular analysis of PLSN‐probes in the size range of 3–5 nm showed an affinity to the cytoskeleton, by which microtubules could be visualized. B) PLSN‐probes ranging from 2 to 3 nm diffuse through permeable cell membranes and adhere to the nucleus/nucleolemma. C,D) The conventional dyes of FITC and DAPI to stain the cytoskeleton (C) and nucleus of cells (D) are shown. Diagnosis of HeLa cells. E) FL image shows the overall view of viable and dead cells (dead and healthy cells are indicated by red and yellow arrows, respectively, in the higher‐magnification FL image on the right). F,G) SEM and FL images demonstrated viable cells (f1, f2) and dead cells (g1, g2). The intensity gradient of the FL images exhibits the dispersion of PLSN‐probes within healthy (f3) and dead cells (g3). H) A comparison of the FL intensities per unit area is shown and indicates a significantly higher intensity from the dead cells. I) Brightfield images taken form control and in presence of the polyhedral PLSN‐probes are shown.

The varied size distribution of the PLSN‐probes not only makes them wavelength‐dependent but also results in a bifunctionality by which different organs of the cell can be differentiated (**Figure** **8**A,B). As demonstrated in Figure 8A, the PLSN‐probes internalized into the cytoskeleton allow one to visualize the microtubules. Since internalization of the PLSN‐probes does not exhibit cytotoxicity or other cellular damage, it is hypothesized that uptake of the PLSN‐probes occurred through the microtubules, as they are components of the cytoskeleton found throughout the cytoplasm. An affinity of the smaller PLSN‐probes to the nucleus inside the cytoplasm was also observed, as shown in Figure 8B. Moreover, as shown in Figure 8C,D, conventional FITC and DAPI dyes, respectively, are used to stain the cytoskeleton and nucleus of cells compared with the PLSN‐probes.

Aside from using the PLSN‐probes for bioimaging purposes, the unique label‐free cellular uptake attributed of these probes has demonstrated their potential use for diagnosing cell health. The diagnostic application of PLSN‐probes relies on the concentration of internalized PLSN‐probes in the cytoplasm, by which the intensity of the emitted light drastically increases. The overall image (Figure 8E) shows the presence of dead and healthy/viable cells. Due to the fact that cytoplasm becomes rounded and shrink toward a cell death, our observations revealed that accumulated PLSN‐probes within a contracted cell (dead cells) display brighter FL.^39^ Therefore, cell shrinkage as a result of cell death was found to be distinguishable through the usage of PLSN‐probes, as displayed in Figure 8E–G. The FL intensity per unit area of dead cells was twofold greater than that of normal (healthy) cells. This offhand diagnostic method is an additional advantage of the synthesized PLSN‐probes. Normalization of the FL intensity per unit area of the cell as a measure to identify the PLSN‐probe uptake provides a means to differentiate dead cells from viable cells (Figure 8F,G). As plotted in Figure 8H, the intensity of the dead cells was almost twice that of the viable cells. The brightfield images (Figure 8I) are in accordance with the ones shown in Figures 5 and 7, where the cells are not only brighter but also more detailed, which as demonstrated can be distinguished at narrow excitation wavelengths. This tool provides a simple method for distinguishing dead from healthy cells. Thus far, organic dyes are the only current imaging means with this function. To the best of our knowledge, a nanoscale, label‐free probe with the capability of screening cellular health has not yet been reported. Of note, this characteristic was found to be dominant in the PLSN‐probes excited at 555 nm or, in other words, the PLSN‐probes ranging in size between 3–5 nm. With the aim of overcoming the aforementioned flaws of organic dyes, two commonly used fluorescent stains, DAPI and FITC, were applied simultaneously with the PLSN‐probes. Figure 8 shows results from the PLSN‐probe‐assisted FL imaging.

**Figure 9:**
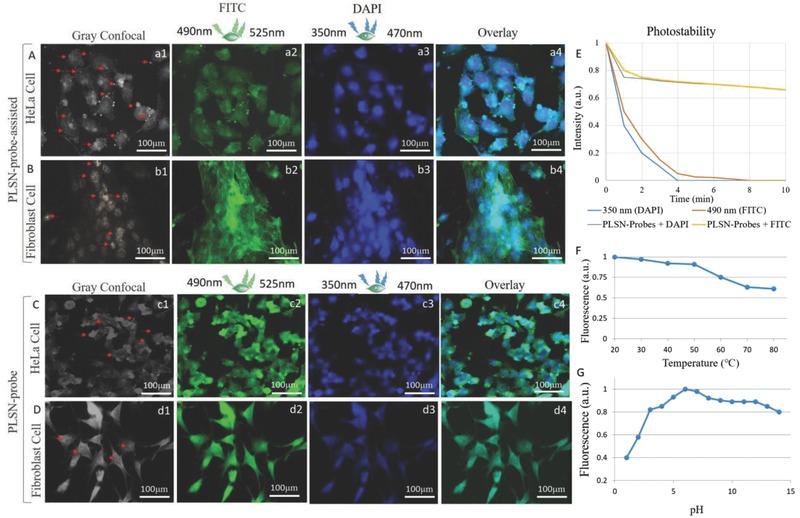
PLSN‐probe‐assisted FL imaging of A) HeLa and B) fibroblast cells and C,D) their counterpart without organic dyes. Confocal imaging of HeLa (a1, c1) and fibroblast (b1, d1) cells shows the uptake of the PLSN‐probes, as indicated by arrow markers. Cells were stained with FITC and DAPI for both cell lines (a2, a3, b2, b3). Overlay of blue and green emissions (a4, b4). The FL imaging of HeLa (C) and fibroblast (D) with PLSN‐probes show gray confocal, excitation at 490 and 350 nm, and overly images, respectively, at (c1, c4) for HeLa and (d1, d4) for fibroblast. E) Photostability (photobleaching) of FITC and DAP in presence and absence of PLSN‐probe are displayed. Fluorescence stability of the PLSN‐probe under variant F) temperature and G) pH. The FL of the PLSN‐probe (F) at different temperatures exhibited descending behavior at around 50 °C. (G) shows alteration of the FL intensity under different pH values (after 6 h).

As numerous studies have proposed, the current QDs used as contrast agents suffer from weak dispersion in aqueous base solutions such as cell culture medium, and hence, effortless dispersion of a contrast agent in cell culture medium is ideal for bioimaging applications.^40^ For PLSN‐probe‐assisted bioimaging, two major excitation wavelengths, 350 and 490 nm, were chosen since blue and green are the most common choices in FL microscopy. The observation revealed higher brightness of the dyes (FITC and DAPI) in the presence of the PLSN‐probes. Moreover, PLSN‐probe‐assisted FL imaging displayed a longer stability of the dyes. **Figure** **9**E depicts the quantitative changes in the photostability of FITC and DAPI compared to that of assisted with PLSN‐probes. DAPI and FITC photobleached after ≈4 min of exposure. However, PLSN‐probe‐assisted FL imaging demonstrated a significantly higher stability of these two dyes. The PLSN‐probe‐assisted FL images in Figure 9A,B, acquired after 10 min of exposure at excitation wavelengths of 350 and 490 nm, still display details of the nucleus and cytoskeleton which are comparable with images in Figure 9(C,D) acquired without using organic dyes.

In addition, the fluorescence stability of the PLSN‐probes was examined at variant pH and temperatures. However, the bioimaging including in vivo and in vitro was carried out at 37 °C. The influence of pH alteration on FL stability of PLSN‐probes were inspected at pH values ranging from (pH = 2, 4, 6, 7, 8, 10, 12), after 6 h, the FL were measured (under a 100 mW 488 and 555 nm continuous wave (CW) excitation wavelengths).^7^, ^41^ The synthesized probes demonstrated slight changes on FL at temperatures higher than 50 °C as well as higher pH stability at biological window (7.2–7.4). The results indicate that the fluorescence stability of the PLSN‐probes remains stable in preferable biological environments.

## 3 Conclusion

Here, we presented a novel approach using polyhedral PLSN‐probes with a broad range of absorption and long Stokes shifts that exhibited multiplex emissions. This label‐free method has shown a precise capability of cell uptake that strongly improves upon the lengthy labeling process of other methods. However, this attribute was found to be cell‐selective, i.e., the HeLa cells demonstrated significantly extended internalization, whereas the fibroblast cells showed a weaker tendency to uptake the PLSN‐probes. The multicrystallinity of the polyhedral structure allows higher absorption for the PLSN‐probe, and hence, a greater FL emission was observed. The PLSN‐probes demonstrated excellent dispersion in aqueous cell culture medium and systematic division into smaller parts that internalize into different organs of the cell, including the nucleus, nucleolemma, and cytoskeleton by which 3D visualization of intercellular organs of the cell is made possible. Moreover, we found that the FL intensity of the PLSN‐probes per cell area increases upon shrinkage of the cytoskeleton of the cell. Hence, this characteristic allows one to conclusively differentiate healthy cells from dead cells. In addition, the proposed probes can be used to diagnose HeLa cells as well as to screen dead cells. Further investigations are required to study the interaction of different cell lines with these PLSN‐probes.

## 4 Experimental Section

To investigate the morphology and dimensions of the synthesized PLSN‐probes, a JEOL JSM‐4800 field emission scanning electron microscope and a JEOL JEM 2100 HR‐TEM were used. HR‐TEM was performed to determine the size distribution and morphology of the PLSN‐probes; for this method, a droplet of suspended PLSN‐probes was placed on a copper grid for direct observation after drying. Image analysis software was used to analyze the HR‐TEM images, perform size measurements, and compile a distribution histogram. An HR‐TEM diffraction pattern (FFT) was also obtained to confirm the formation of crystalline PLSN‐probes. Energy‐dispersive X‐ray spectroscopy (EDX) analyses using Oxford Instruments further corroborated the formation of pure PLSN‐probes and the elemental mapping evidenced the self‐internalization of the synthesized PLSN‐probes into the cells. The Raman spectra were recorded with a Bruker SENTERRA dispersive Raman microscope using a wavelength of 325 nm. The Raman spectra showed a strong band and sharp peaks at 521 cm^−1^, which are characteristics of crystalline PLSN‐probes. UV–vis–NIR absorption and reflection spectra were obtained with a Hitachi UV‐3100 UV–vis–NIR spectrophotometer. PL spectra were recorded on a Shimadzu RF‐5301PC spectrofluorophotometer at room temperature.

*Synthesis of PLSN‐Probes*: A single‐crystal N‐type silicon wafer with a (100) crystallographic was sliced into 2 cm^2^ square‐shaped samples, which were ultrasonically cleaned in a 50 °C acetone bath for 15 min, followed by rinsing and drying. To synthesize the PLSN‐probes, silicon substrates were exposed to a diode‐pumped Yb‐doped femtosecond laser beam (Clark‐MXR, Inc., IMPULSE Series ultrashort pulse laser). This laser system is capable of producing central wavelengths of 1040 nm with a pulse width that can be varied, ranging from 214 to 1428 fs, as well as a tunable laser pulse repetition rate that can vary between 4 MHz (low rep. rate) and 26 MHz (high rep. rate). A maximum average operating power of 16 W was attainable. Utilizing the ultrashort (femtosecond range) pulsed laser is advantageous since the absorption of multiphoton excitation energy delivers the required energy of ionization and also, given the localized laser beam spot within the focal point, results in precise control over the synthesized PLSN‐probes. The samples were, therefore, irradiated at the beam focal point, with a computerized galvano scanner precisely implementing the synthesis processes. The self‐assembled PLSN‐probes were synthesized by single‐step femtosecond laser processing of the silicon substrate. For the purpose of this study, the polyhedron, intermediate, and polyhedral PLSN‐probes were fabricated with a pulse width of 214 fs and repetition rates of 4, 12, and 26 MHz, respectively. The laser fluences at 4, 12, and 26 MHz were measured as 1.57, 3.39, and 10.18 J cm^−2^, respectively (the laser power was 16 W, with a Gaussian 1/e^2^ beam diameter of 10 µm, and the scanning speed was 2 mm s^−1^; all parameters were held constant.) To ensure reproducibility of the synthesized PLSN‐probes, the synthesis method was performed three times at the given conditions. Figures S1–S3 (Supporting Information) further illustrate average particle size distributions and HR‐TEM images of the polyhedral and polyhedron PLSN‐probes.

*Cell Culture and Seeding*: The synthesized PLSN‐probes were sterilized under UV light for 20 min prior to their addition to cell culture medium. Fibroblasts (NIH3T3) and human cervical cancer cells (HeLa, ATCC, American Type Culture Collection, ATCC No. CCL‐2) were employed in cell experiments to ascertain the comparative functionality of mammalian and cancer cell lines in response to the samples. Fibroblast cells were grown in Dulbecco's modified Eagle's medium (DMEM) containing 10% heat‐activated fetal bovine serum with 1% penicillin–streptomycin antibiotics (Pen‐strep). HeLa cells were grown in DMEM‐F12 supplemented with 10% fetal bovine serum and 1% Pen‐strep. Subsequently, the cells were separately cultured on substrates placed in Petri dishes with a seeding density of 750 000 cells cm^−2^. The Petri dishes were placed in an incubator for 24 and 48 h at 37 °C. Following the PLSN‐probe cell response study, the cells were studied via scanning electron microscope (SEM) and confocal FL microscopy.

*Cell Imaging*: The morphology of the cells was observed using a SEM (Hitachi, SU1510). For this aim, after the prescribed time period, the spent medium was aspirated. The samples were then fixed in 2% glutaraldehyde in (0.1 m) pH 7.3 sodium cacodylate buffer for 1 h. Next, the samples were dehydrated at increasing concentrations of alcohol for 20 min, followed by immersion in (0.1 m) sodium cacodylate buffer with (0.2 m) pH 7.3 sucrose for 20 min. The samples were then critical point dried. After the experiment, the cells were prepared for direct observations via SEM.

*Fluorescence Microscopy*: For FL microscopy, the samples were first fixed in methanol‐free paraformaldehyde followed by incubation in skim milk to prevent nonspecific binding. To stain the actin and cytoskeleton, the samples were incubated with Alexa Fluor 488 (Life Technologies) followed by DAPI (Life Technologies) to stain the nucleus. The samples were studied using a FL microscope (Nikon, Canada). Seeded cells were also fluorescence‐imaged on a Zeiss LSM 710 META upright confocal laser‐scanning microscope using 60× magnification water‐dipping lenses for the monolayer cultures. Wavelengths of 405, 488, 555, and 633 nm were used. Image data acquisition and processing were performed using a Zeiss LSM Image Browser, Zeiss LSM Image Expert and ImageJ.

*Phosphorescence Lifetime and Intensity Measurement*: Photoluminescence lifetime imaging was performed on a fluorescence microscope (Zeiss LSM 710 META) equipped with confocal laser scanning system. The synthesized PLSN‐probes were excited at 405, 488, 555, and 633 nm and detected through center wavelength of 447, 525, 617, and 635 nm and bandwidth of 60 nm, 45 nm, 36 nm, and longpass, respectively.

*Statistics*: All experiments were performed in triplicate, and the data points were averaged unless otherwise indicated. The error bars show standard deviations.

## Conflict of Interest

The authors declare no conflict of interest.

## Supporting information

Supplementary
