# Supplementary material for: Multiplex Photoluminescent Silicon Nanoprobe for Diagnostic Bioimaging and Intracellular Analysis
Source: Adv Sci (Weinh). 2017 Dec 31;5(3):1700548. doi: 10.1002/advs.201700548 (PMC5867044; doi:10.1002/advs.201700548)
Supplement: Supplementary file 1 — Supplementary [file ADVS-5-1700548-s001.pdf]

Copyright WILEY-VCH Verlag GmbH & Co. KGaA, 69469 Weinheim, Germany, 2017.

Supporting Information

for *Adv. Sci.,* DOI: 10.1002/advs.201700548

Multiplex Photoluminescent Silicon Nanoprobe for Diagnostic Bioimaging and Intracellular Analysis

*Meysam Keshavarz, Bo Tan, and Krishnan Venkatakrishnan**

Copyright WILEY-VCH Verlag GmbH & Co. KGaA, 69469 Weinheim, Germany, 2017.

Supporting Information

Multiplex Photoluminescent Silicon Nano-Probe for Diagnostic Bioimaging and Intracellular Analysis

*Meysam Keshavarz, Bo Tan, and Krishnan Venkatakrishnan **

Supporting Information

The following sets of results were obtained to ensure reproducibility of the synthesized PLSN-probes and their subsequent application for bioimaging.

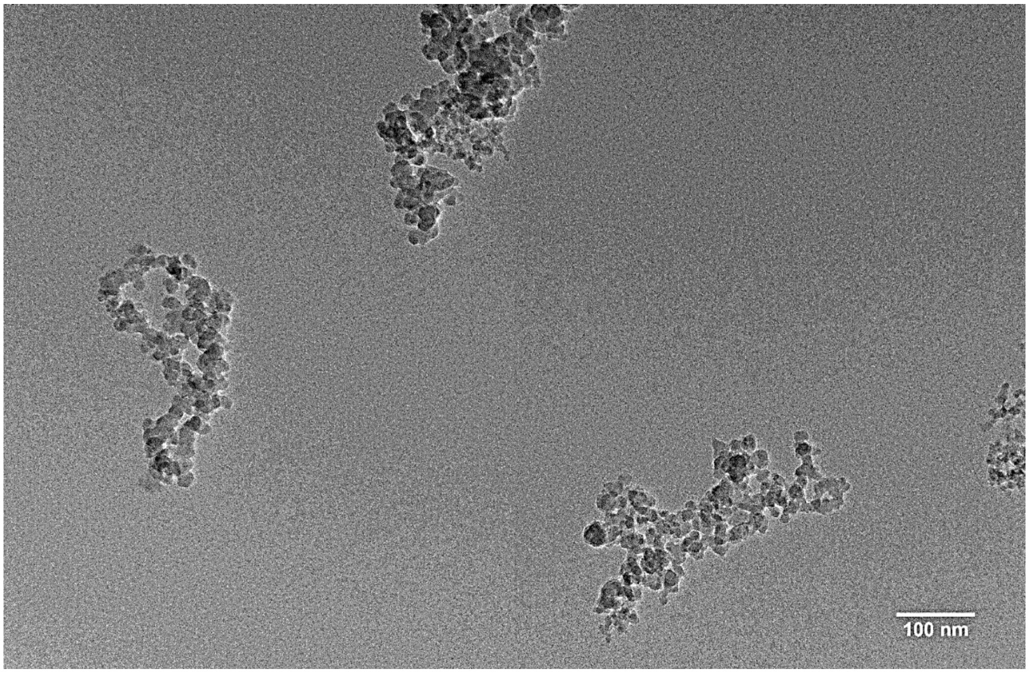

Figure S1. HR-TEM micrographs shows the morphological and an average particle size distribution of the synthesized PLSN-probes.

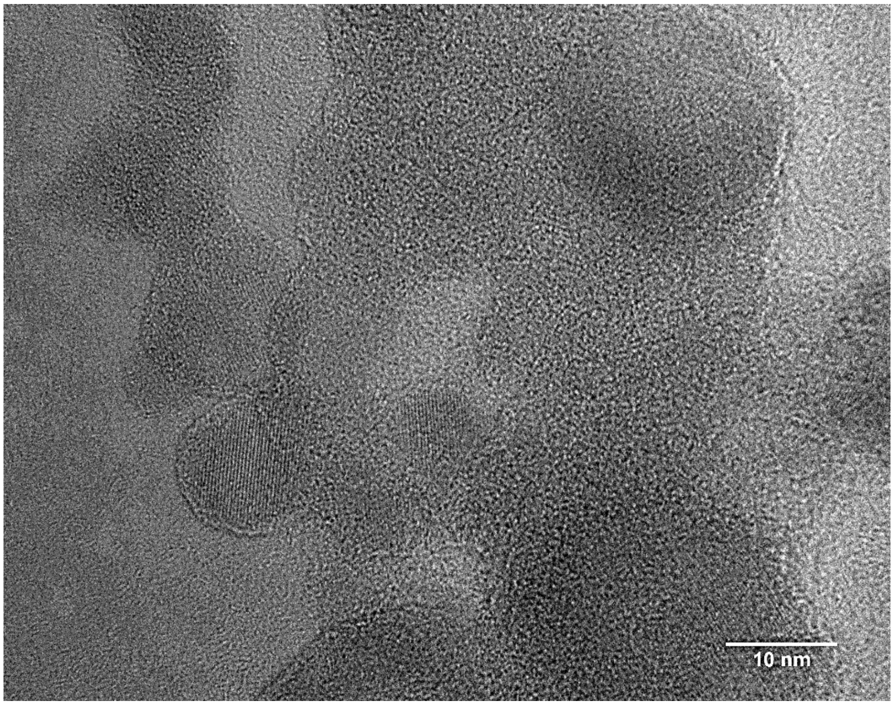

Figure S2. HR-TEM micrograph shows the crystal plane orientation of the polyhedron PLSN-probes.

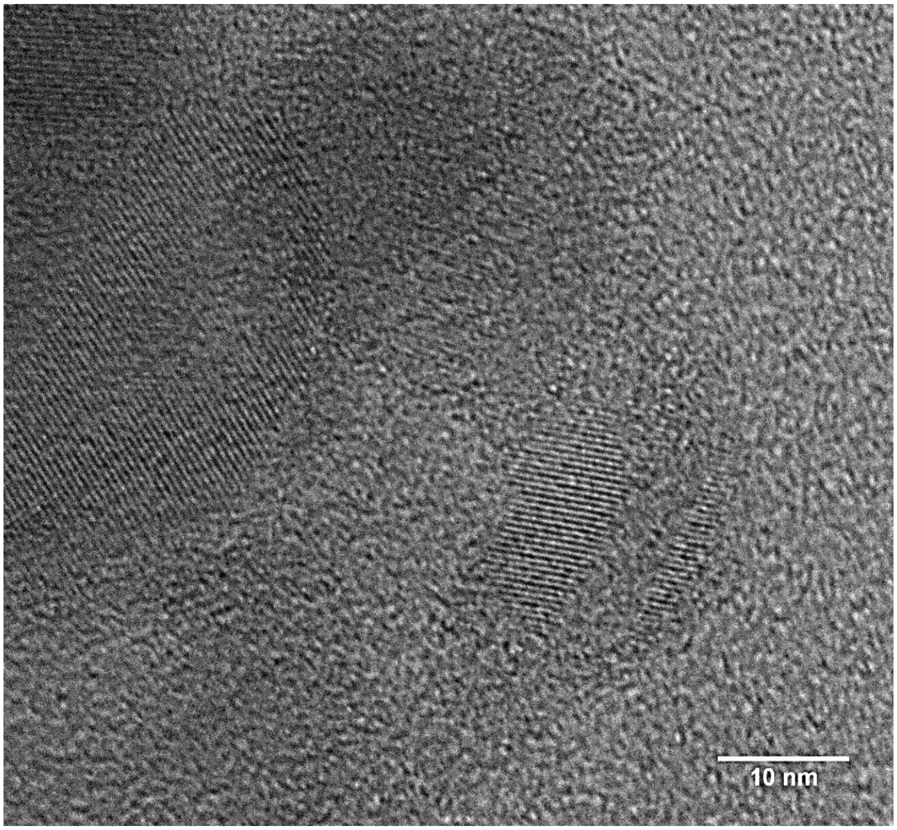

Figure S3. HR-TEM micrograph shows the crystal plane orientation of the ployhedral PLSN-probes.

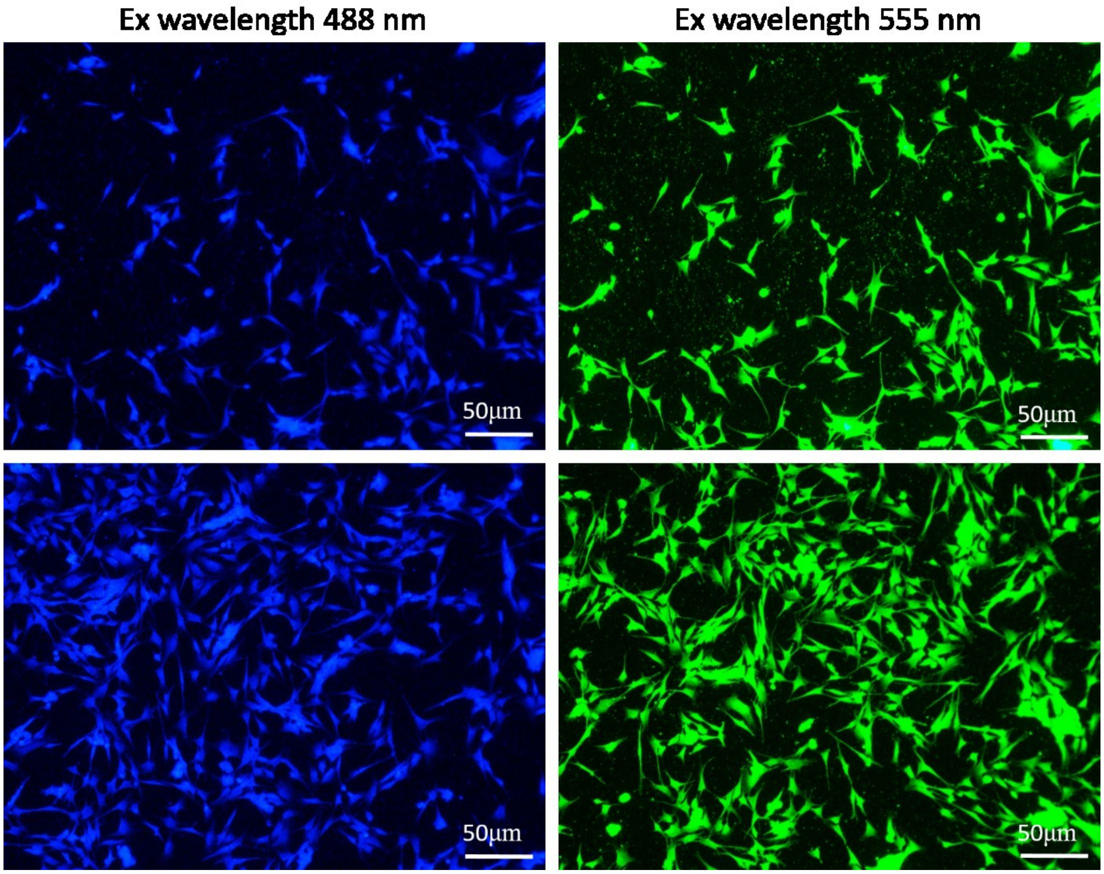

Figure S4. Confocal microscopy of fibroblast cells acquired (2 separate sets of experiments) at 448 and 555nm excitation wavelengths demonstrate disinclination of the fibroblast cells to uptake the PLSN-probes.

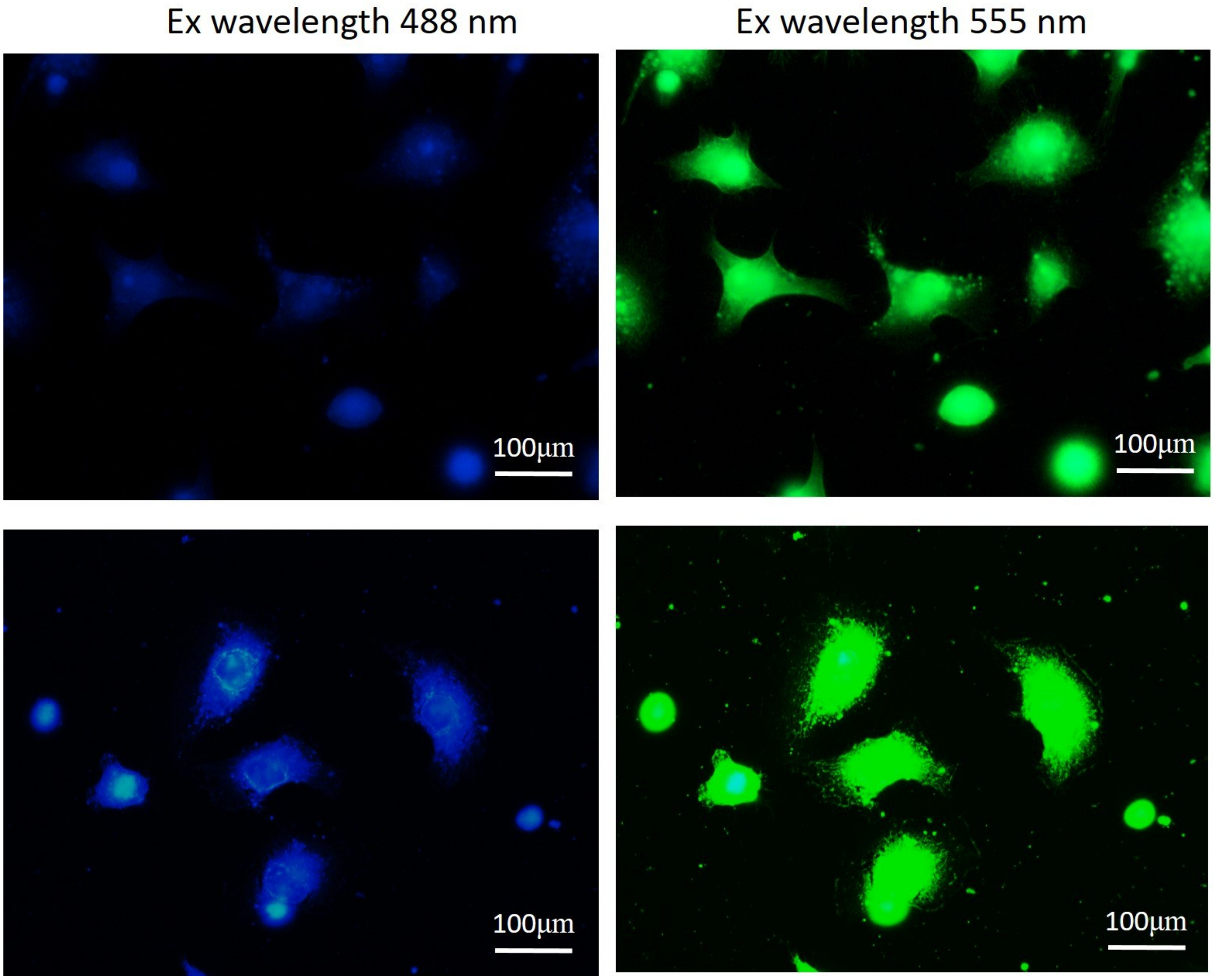

Figure S5. Confocal microscopy of HeLa cells acquired (2 separate sets of experiments) at 448 and 555nm excitation wavelengths demonstrate tendency of the HeLa cells to uptake the PLSN-probes.
